# Effect of chitosan and chlorhexidine on demineraslised dentin to enhance adhesion

**DOI:** 10.6026/973206300210185

**Published:** 2025-02-28

**Authors:** Rashmi P Yadahalli, Saikiran Bahadur, Nisha Gupta, Smita Singh Bhardwaj, Sushree Arpita Priyadarsani, Ahmed Ali Ahmed Almuntashiri, Awadhesh Kumar Gupta

**Affiliations:** 1 Department of Conservative Dentistry and Endodontics, PMNM Dental College & Hospital, Bagalkot, Karnataka, India; 2 Department of Prosthodontics, Springfield Dental, Springfield, Massachusetts, USA; 3 Department of Conservative Dentistry and Endodontics, ESIC Dental College and Hospital, Delhi, India; 4 Department of Preventive Dental Sciences, College of Dentistry, Majmaah University, Al Majmaah-11952, Saudi Arabia; 5 Intern, Kalinga Institute of Dental Sciences, Bhubaneswar, Odisha, India; 6 Department of Public health, College of Applied Medical Sciences, Qassim University, Buraydah, 51452, P.O. Box 6666, Saudi Arabia; 7 Department of Oral Pathology and Microbiology, DJ College of Dental Sciences and Research, Modinagar, Ghaziabad, Uttar Pradesh, India

**Keywords:** Adhesion, chitosan, chlorhexidine, demineralised, dentin, infection

## Abstract

The bond strength (BS) and failure status of demineralized dentin following the application of chitosan solution and chlorhexidine (CHX) is of interest to dentists. A total of 30 non-pathological extracted premolar teeth were subjected to caries induction through the pH cycle procedure. The teeth were divided equally into 3 groups as such as group I: control (distilled water), Group II-2.5% chitosan solution and Group III - chlorhexidine (CHX). Later teeth were subjected to a microtensile bond strength test (µTBS). The failure mode was assessed using stereo microscope. The bond strength of the chitosan-treated specimens was significantly higher than that of the chlorhexidine treated specimens and the control specimen which had the lowest bond strength.

## Background:

Composite restorations have become widely used in restorative dentistry over the past two decades as a result of advancements in the material's adhesive properties and the material itself [1]. To achieve bond strength to dentin, adhesive systems, total-etch approaches can be employed [2]. Minimally invasive dentistry prioritizes the prevention and before time interference of caries with minimal restoration and preservation. The efficacy of this treatment is to predict the conception of careful elimination of caries tissue [3]. Nevertheless, the development of a bio-adhesive boundary in a partially demineralized substrate requires attention due to the disorganized organic matrix and distinct morphological characteristics of dentin affected by caries [4]. Dentin is a complex tissue that is made up of minerals, water and organic components, including collagen. The bonding outcome among dentin and composite resin is not as strong as that of enamel due to the structural characteristics of dentin. A diverse display of matrix metallo-proteinases (MMPs) and cysteine catharsis are present in dentin, typically in the form of zymogen. The constancy and permanence of the dentin bonding interface have consistently been a pressing issue in the field of adhesive dentistry [5]. Certain constituents may be included into adhesive systems or restorative materials to prevent the passage of free monomers in the direction of the pulp or to reduce the damage they cause. An example of these components is chitosan [6]. In numerous fields of dentistry and medicine, the utilization of chitosan extracts has been emphasized [7]. Chitosan is a hydrophilic polysaccharide that is produced through the de-acetylation of chitin, the second most abundant biopolymer in nature. Chitosan has been employed in dentistry as a gel [2, 8].

Chitosan is a biopolymer that is naturally present in the cell walls of fungi, yeasts, insects and most notably, crustaceans' shells. It is produced through the de-acetylation of chitin. Chitosan possesses a variety of advantageous characteristics, including biocompatibility, hydro-philicity, biodegradability, non-toxicity and bio-adhesiveness [9, 10]. In addition to its antibacterial properties, it is an antioxidant and antifungal that inhibits collagen matrix degradation [11]. Because of its amino groups and the formation of cross-links with dentin collagen, the chitosan molecule enables substitution reactions in a chemical sense. Its adhesiveness is the consequence of electrostatic bonding, in which the collagen carboxyl group (COO- attracts the chitosan amine group (NH3+) [9]. Consequently, chitosan is employed in the field of restorative dentistry. Chitosan may be integrated into a variety of restorative materials, including glass ionomers cement [10]. Chlorhexidine is a nonspecific inhibitor of MMPs that has the ability to inhibit MMP-2, MMP-8 and MMP-9.66the stability of the dentin bonding interface can be maintained by pre-treatment of dentin with chlorhexidine, which can protect collagen in the mixed layer from degradation by MMPs [12, 13]. Collapse fiber, dentin collagen exposure and resin monomer infiltration are all adversely affected by adhesion to the demineralized and dried dentin surface [14]. Dentin bio-modification is crucial for enhancing the structural stability of the dentin collagen matrix. Therefore, it is of interest to assess the bond strength (BS) and failure mode of demineralized dentin following the application of chitosan and chlorhexidine solution.

## Materials and Methods:

A total of 30 non-pathological extracted premolar teeth that were indicated for orthodontic purposes were chosen. The roots were sectioned 1 mm below the cemento-enamel junction using a precision cutter-coupled diamond disc and the occlusal enamel was removed. Thirty teeth were subjected to caries initiation using the pH cycle procedure, which involved immersing the teeth in 10 mL of demineralizing solution for 8 hours, subsequently re-mineralization for 14 days. The careful elimination of decayed tissue was performed using carbide drills. Subsequently, these teeth were alienated into three groups: Group I (Control), Group II (2.5% chitosan solution) and Group III (chlorhexidine (CHX). In the second and third test groups, chitosan solution was actively applied to the dentine surface for one minute, followed by drying with absorbent paper. Control group specimens were not treated. Adhesive and composite resins were used to restore the tooth surfaces in accordance with the manufacturer's instructions. The teeth specimens were then sectioned, with one half remaining untreated and the other half subjected to aging. The aging process involved 12,000 thermal cycles, enzymatic degradation and 6 months of storage in water. Subsequently, the universal testing machine was employed to conduct a microtensile bond strength test (µTBS) on both halves. At a crosshead speed of 0.5 mm/min and a load of 50 kg/f, the specimens were subjected to tension from the device's extremities until they failed. The failure pattern of each fractured specimen was categorized as adhesive when it occurred at the resin-dentin interface and as cohesive of material when the surface was wholly covered by composite resin by analysing both halves under a stereomicroscope (Nikon, Melville, NY, USA). Data analysis was performed using ANOVA test at p<0.05.

## Results:

The values of sound dentin were considerably greater than those of demineralized dentin (p < 0.001). Table 1 indicates that the bond strength of the specimens treated with 2.5% chitosan solution was substantially higher followed by chlorhexidine and the least with the untreated control specimens (p < 0.001). Addition of chitosan and chlorhexidine had no considerable influence on the failure mode (Table 2).

## Discussion:

Chitosan has been identified as a critical biomaterial that prevents the degradation of the dentin organic matrix by metalloproteinase by stabilizing the adhesive interface through the formation of crosslinks with collagen fibrils [15]. The current research assessed the efficacy of chitosan as a method for preserving the hybrid layer, which enables the infiltration of resin monomers into the interfibrillar spaces of the dentin collagen matrix. This process is accountable for the micromechanical retention of the restorative material on the substrate. The sound specimens exhibited greater bond strength values than the demineralized specimens, as indicated by the analysis. Chitosan's capacity to interact with the dental structure was demonstrated by its association with increased bond strength values in specimens. Significant reductions in bond strength values were observed in the control group following thermal cycling. The failure mode was not considerably impacted by the addition of chitosan and chlorhexidine, as we discovered. Our results are consistent with those of other studies. Ziotti *et al.* discovered that demineralized dentin exhibited enhanced bond strength following aging when treated with chitosan [3]. Nunes *et al.* concluded that the bond strength and failure mod were unaffected by the addition of 0.2 or 0.5% of chitosan [2]. The study conducted by Paschoini *et al.* concluded that the treatment of dentin with chitosan in conjunction with an etch-and-rinse or self-etch adhesive system resulted in an improvement [16]. Zhao *et al.* assessed the durability of resin-dentin bonding interfaces by pre-treatment of dentin with chitosan-loaded oleuropein nanoparticles (CONPs). The author concluded that CONPs had the potential to function as a dentin precursor, which could significantly enhance the durability of dentin-resin binding. After thermocycling, chlorhexidine and CONP exhibited higher tensile bond strength values [5]. Abdul-Razzaq *et al.* assessed the macro shear bond strength of resin composites through the use of chitosan nanoparticles and NAF solutions for dentin surface pre-treatment. They determined that the shear bond strength of the etch-and-rinse adhesive system was not substantially impacted by dentin pre-treatment with 0.2% chitosan solution [10]. Halkai *et al.* discovered that the bond strength was not adversely affected by the incorporation of chitosan nanoparticles (CSN) in composite or dentin bonding agent (DBA) [1]. Surmelioglu *et al.* conducted a comparison of the bond strength of teeth treated with radiotherapy and two cavity disinfectants (Chlorhexidine gluconate, a chitosan-containing agent). They found that the bond strength was negatively impacted by radiotherapy, while the use of disinfectant agents had a positive impact [11]. In contrast to our findings, Stenhagen *et al.* assessed the impact of methacrylate chitosan added to experimental adhesives and found that there were no changes in dentin's binding strength [17]. According to El-Din *et al.* adding nano-chitosan at 0.5% and 1% can improve the material's universal adhesive microtensile bond strength and bond durability [18]. In order to improve bond durability, Gu *et al.* evaluated the possibility of employing chitosan as an antibacterial extra-fibrillar dentin-chelating agent. They came to the conclusion that chitosan had bactericidal properties against three single species, preserved intra-fibrillar minerals and increased the endurance of the resin-dentin bond [19]. A smaller sample size and in vitro examination were the study's limitations.

## Conclusion:

The bond strength of demineralized dentin was enhanced by chitosan treatment following aging. Further, failure mode was not impacted by the addition of 2.5% chitosan and chlorhexidine.

## Figures and Tables

**Table 1:** Micro tensile bond strength of dentin in both control and test group

**Dentin type**		**Groups**		**p**
	**Group I**	**Group II**	**Group III**	
Sound	30.32 ±4.76	31.42 ± 7.64	31.18 ± 5.54	0.563
Demineralised	8.65 ± 3.86	14.84 ± 4.24	10.73 ± 4.32	0.001
P	0.001	0.001	0.001	

**Table 2:** Failure patterns (%) among different groups

**Groups**	**Adhesive Fracture**	**Dentinal cohesive fracture**	**cohesive fracture in resin**
Group I	16%	85%	13%
Group II	10%	76%	12%
Group III	12%	69%	10%
